# Anxiety and Stress Related to COVID-19 Among the Community Dwelling Older Adults Residing in the Largest Refugee Camp of the World

**DOI:** 10.1007/s10597-023-01101-5

**Published:** 2023-03-06

**Authors:** Afsana Anwar, Uday Narayan Yadav, Md. Nazmul Huda, Sukanta Das, Simon Rosenbaum, A. R. M. Mehrab Ali, Probal Kumar Mondal, Abu Ansar Md. Rizwan, Syed Far Abid Hossain, Suvasish Das Shuvo, Sabuj Kanti Mistry

**Affiliations:** 1 Health and Nutrition, Social Assistance &Amp; Rehabilitation for the Physically Vulnerable (SARPV), SARPV Complex, Link Road, Cox’s Bazar, 4700 Bangladesh; 2 National Centre for Epidemiology and Population Health, The Australian National University, Canberra, Australia; 3 Centre for Primary Health Care and Equity, University of New South Wales, Sydney, Australia; 4 School of Medicine, Translational Health Research Institute, Western Sydney University, Campbelltown, NSW 2560 Australia; 5 Department of Statistics, Begum Rokeya University, Rangpur, Bangladesh; 6 School of Psychiatry, University of New South Wales, Sydney, Australia; 7 ARCED Foundation, 13/1, Pallabi, Mirpur-12, Dhaka, Bangladesh; 8 BRAC Business School, BRAC University, Dhaka, Bangladesh; 9 Department of Nutrition and Food Technology, Jashore University of Science and Technology, Jashore, Bangladesh; 10 Department of Public Health, Daffodil International University, Dhaka, 1207 Bangladesh; 11 Brain and Mind Centre, The University of Sydney, Sydney, Australia

**Keywords:** Rohingya, Older adults, COVID-19, COVID-19-related anxiety, Perceived stress

## Abstract

The current cross-sectional study was conducted among 864 older adults aged ≥ 60 years residing in Rohingya refugee camp through face-to-face interviews during November–December 2021. COVID-19-related anxiety was measured using the five-point Coronavirus Anxiety Scale (CAS) and perceived stress using the 10-point Perceived Stress Scale (PSS). The linear regression model identified the factors associated with COVID-19-related anxiety and perceived stress. The prevalence of COVID-19-related anxiety and perceived stress was 68% and 93%, respectively. The average COVID-19-related anxiety score expected to be significantly higher among those who were physically inactive, concerned about COVID-19, had a close friend/family member diagnosed with COVID-19, and had some difficulty in getting food and routine medical care during the COVID-19 pandemic. Meanwhile, the average perceived stress score was expected to be significantly higher among those without partners, who were feeling overwhelmed by COVID-19, and who experienced COVID-19-related anxiety during the pandemic. The findings suggest providing immediate psychosocial support to older Rohingya adults.

**Supplementary Information:**

The online version contains supplementary material available at 10.1007/s10597-023-01101-5.

## Introduction

The coronavirus disease 2019 (COVID-19) pandemic and accompanying control measures created major interruptions to social norms and public health practices worldwide (Chadi et al., 2022). According to the World Health Organization (WHO), as of 17 November 2022, over 632 million confirmed cases of COVID-19 had been identified, and over 6.5 million deaths had occurred due to the pandemic (World Health Organization, 2022c). Evidence shows that COVID-19 pandemic not only hampered the physical health of the diseased, but also affected the mental well-being of the patients and the community as a whole (Knolle et al., 2021; Pfefferbaum & North, 2020). This has been a particular concern for the vulnerable populations such as older adults, refugees and people with long term conditions (Yadav et al., 2020).

Emerging evidence during the COVID-19 pandemic shows that mental health outcomes, including anxiety, stress and depression, affected almost everybody globally (Mistry et al., 2022; Robinson et al., 2022; Xiong et al., 2020). The COVID-19 pandemic exerted a detrimental psychological impact at both individual and community levels (Salari et al., 2020). The WHO has stated that the global prevalence of anxiety has increased by 25% during the COVID-19 pandemic (World Health Organization, 2022b). Recent systematic reviews also reported that the pooled prevalence of anxiety and stress to be 31.9% and 29.6% respectively in general population amid COVID-19 pandemic (Mahmud et al., 2022; Salari et al., 2020). Unplanned lockdowns and difficulties in accessing essential services, including food, health care and medication, as well as social distancing and isolation as preventive measures have contributed to increased anxiety and stress during the pandemic (Cheruvu & Chiyaka, 2019; Tausch et al., 2022). Notably, higher COVID-19 related anxiety also found to be positively associated with increased stress during the COVID-19 pandemic (Hu et al., 2021). While everyone is at risk of adverse mental health conditions during the pandemic, older adults are particularly vulnerable (Vahia et al., 2020; Webb & Chen, 2022). Pre-existing medical health conditions (e.g. respiratory diseases, hypertension, chronic kidney diseases, and obesity), limited financial and social support, limited access to resources and emergency services make older people more vulnerable to developing mental illnesses during the COVID-19 pandemic (Lee et al., 2020).

Worsening of mental health conditions during the COVID-19 pandemic can be a particular concern among the displaced and refugee population, partly because of their unsuitable living condition, lack of access to health services, material deprivation, isolation, and uncertainty in their life (Anwar et al., 2022; Júnior et al., 2020; Singh et al., 2018; Spiritus-Beerden et al., 2021). Evidence showed that the prevalence of mental health problems among refugee population varied from 20% to 80% globally (Júnior et al., 2020). The Rohingyas are a Muslim minority from Myanmar, mainly seek refuge in Bangladesh since the seventies due to denial of citizenship from the concerned authority from Myanmar, but have also fled to other countries such as Saudi Arabia, Pakistan and Malaysia (Alam 2019). A total of 943,529 people are currently living in Rohingya camp in Cox’s Bazar, a Southern district of Bangladesh, and is the largest refugee camp in the world (United Nations High Commissioner for Refugees, 2022). Older adults comprise a significant portion of the Rohingya community and recent records suggest that older people comprise 3.6% of the total population residing in the camp (United Nations High Commissioner for Refugees, 2022). Rohingya camp is densely populated, with 40,000 people living per square kilometer and lack access to basic amenities such as safe water, satiation and health facilities (Kamal et al., 2020). COVID-19 has also been a particular concern in the Rohingya camp as there were 6,793 confirmed COVID-19 cases and 45 deaths till 17 November 2022 (World Health Organization, 2022a).

Age being a crucial factor for emotional distress, the impact of COVID-19 on aggravating anxiety and stress among the older adults residing in the camps can be serious. A longitudinal study conducted on the Rohingya population showed that the stress level was significantly increased during the COVID-19 pandemic compared to that of the pre-pandemic rate (Palit et al., 2022). However, the participants of this study were younger adults. Another study conducted among older adults aged 60 years and above residing in the Rohingya refugee camp demonstrated a high prevalence of depressive symptoms during the COVID-19 pandemic (Mistry et al., 2021b). However, to the best of our knowledge, no study has explored the prevalence of anxiety and stress during the pandemic among the older adults living in the Rohingya camp in Bangladesh. Therefore, the current study aimed to (i) examine the prevalence of COVID-19-related anxiety and perceived stress among the community dwelling older Rohingya adults and (ii) identify the factors associated with COVID-19-related anxiety and perceived stress among them.

## Methods

### Study Design and Participants

This study followed a cross-sectional design conducted between November and December 2021. Participants were older adults aged 60 years and above residing in five purposively selected sub-camps of Rohingya refugee camp in Bangladesh where Social Assistance and Rehabilitation for the Physically Vulnerable (SARPV) is currently working.

Considering an unknown prevalence of anxiety/stress with a 5% margin of error, 95% level of confidence, 80% power of the test and 25% non-response rate, the required sample size was calculated as 973. Finally, a total of 864 participants consented to take part in the study from the approached 973 participants (response rate 88.8%). The SARPV had a list of all participants residing in the five selected sub-camps (of the 34 sub-camps in the Rohingya camp) which were used as the sampling frame for the study. A simple random sampling technique (computer generated numbers) was used to select the required number of participants. Age of the beneficiaries were verified using SMART card provided by UNHCR containing all relevant information. The inclusion criteria were aged 60 years or above and residents of Rohingya camps. The exclusion criteria were presence of any adverse mental conditions (clinically diagnosed schizophrenia, bipolar mood disorder, dementia/cognitive impairment), a hearing disability, or an inability to communicate.

## Measures

### Outcome Measure

COVID-19-related anxiety was measured using the Bengali version of the five-point Coronavirus Anxiety Scale (CAS) (Ahmed et al., 2020), which was translated to Rakhine language (commonly used by Rohingya people). Participants were asked about their level of COVID-19-related anxiety, experienced in the last two weeks before the survey using the CAS and their agreement/disagreement with five CAS items were assessed using a five-point Likert Scale. Therefore, the cumulative score ranged from 0 to 20, where the higher the scores, the greater the anxiety of COVID-19. We further classified the participants as having COVID-19-related anxiety (if they reported having anxiety in any one of the CAS items) or not having COVID-19-related anxiety (if they reported they have had no anxiety in any CAS item). We found the reliability of the scale among the participants acceptable (Cronbach’s α = 0.84).

Similarly, perceived stress was measured using the 10-items Perceived Stress Scale (PSS), which was previously validated among Bangladeshi population (Islam, 2020). This tool was translated to the Rakhine language and participants were asked if they perceived stress in the last month preceding the survey using the PSS-10 and their agreement/disagreement with the ten items were assessed using a five-point Likert Scale. Therefore, the cumulative score ranged from 0 to 40, where higher scores indicated greater levels of perceived stress. We further classified the participants as having perceived stress (if they reported having stress in any one of the PSS items) or not having perceived stress (if they reported they have had no stress in any PSS item). Reliability of the scale was acceptable (Cronbach’s α = 0.79).

### Explanatory Variables

Explanatory variables considered in this study were selected based on extensive literature review (Huda et al., 2021; Lou et al., 2012; Mistry et al., 2021b; Perez et al., 2001; Renner et al., 2021; Stubbs et al., 2017; Tinghög et al., 2017). We considered age (categorized as 60–69, and ≥ 70), sex (male/female), marital status (married/without partner), formal education (yes/no), household size (≤ 4 or > 4), monthly family income in Bangladeshi Taka (BDT) where 1 USD ~ 90 BDT (Living on aid alone, have some additional income, current occupation (employed/unemployed or retired), living arrangements (living alone or with family), walking distance to the nearest health centre (< 30 min/ ≥ 30 min), currently suffering from any non-communicable chronic diseases (NCDs) (yes/no), level of physical activity (regular at least 2–4 h per week/none or sedentary), feeling concerned about COVID-19 (hardly, sometimes/often), feeling overwhelmed by COVID-19 (hardly, sometimes/often), close friend or family member previously diagnosed with COVID-19 (no or not sure/yes), frequency of communication with friends and family during COVID-19 (less than previous/same as previous), difficulty in obtaining food, earning money and getting routine medical care during COVID-19 (no/yes) as explanatory variables in the study.

The median household size of the Rohingya population residing in the camp is 4.0 (Bhatia et al., 2018). Therefore, we categorized the household size as ≤ 4 or > 4. Self-reported information on sufferings from chronic conditions (e.g., arthritis, hypertension, heart diseases, stroke, hypercholesterolemia, diabetes, chronic respiratory diseases, chronic kidney disease, and cancer) was collected. Thereafter, a new variable was created, “currently suffering from any non-communicable chronic diseases (NCDs)” which was categorized as “No” if they did not have any of these diseases and “Yes” if they had at least one of these diseases. In line with the previous literature, we categorised the walking distance to the nearest health centre as less than 30 min and equal to or more than 30 min (Mistry et al., 2021b).

### Data Collection Tools and Techniques

A pre-tested semi-structured questionnaire in the Rakhine language was used to collect the information through face-to-face interviews. Data were electronically recorded in SurveyCTO mobile app (https://www.surveycto.com/) by two enumerators, who were fluent in Rakhine dialects. They had previous experience in administering health surveys using electronic data collection platforms. The enumerators were trained for three days before commencing data collection, including on procedures of maintaining COVID-19 safe behaviours during the data collection.

The Bengali version of the questionnaire was first translated to Rakhine dialects and then back-translated to Bengali by two staff of SARPV who understand both Bengali and Rakhine language. The Rakhine version of the tool was piloted among a small sample (n = 10) of older Rohingya adults from the selected camps to refine the language in the final version. The participants approved the tool translated into Rakhine language without any corrections or modifications. Data collection was accomplished using this final tool through face-to-face interviews of participants. Each interview took approximately 30 min.

### Statistical Analysis

The distribution of the variables was assessed through descriptive statistics. Variables were checked for missing values, and none of the outcome and explanatory variables had any missing values. Two separate multiple linear regression models were performed to explore the factors associated with COVID-19-related anxiety and perceived stress among the participants. All independent variables were examined for multicollinearity before entering them in the regression analysis, and no significant multicollinearity between any independent variables was identified. We have also checked whether the dependent variables met the assumptions for linear regression and found that they met the assumptions including that they are normally distributed. Thereafter, a backward elimination criterion with the Akaike Information Criterion (AIC) was employed to select the final model. Briefly, the backward elimination algorithm starts with a full model (model with all variables) and drops one by one variable from the model based on the statistical significance of that variable. In this case, the adjusted beta coefficient, p-value, and 95% confidence interval (95% CI) for the final multiple linear regression analysis model are reported in the main table, and the multicollinearity diagnostics results are presented in a separate table. All analyses were performed using the statistical software package Stata (Version 17.0).

## Results

### Characteristics of the Participants

A total of 864 participants aged 60 years and over participated in this study. Table 1 shows the socio-demographic characteristics of the participants as well as their perceived opinion on COVID-19-related information. More than half of the participants (57%) were males. The majority of the participants were aged 60–69 years (72%), currently married (79%), had no formal schooling (89%), lived with family members or others (91%), was living on aid alone (67%), and were currently unemployed or retired (89%). Around half of the participants had large household size with more than 4 members (57%) and were currently suffering from any non-communicable chronic diseases (NCDs) (50%). Nearly one-third of the participants (31%) resided more than 30 min walking distance from the nearest health center, and more than two-thirds (66%) did not engage in regular physical activity. In terms of perceived opinion on COVID-19-related information, most participants were somewhat to very concerned (80%) and felt overwhelmed (78%) by COVID-19. About 8% of the participants’ close friends or family members were previously diagnosed with COVID-19. More than a quarter of the participants (29%) reported that the frequency of communication during COVID-19 was less often than previous. Most participants reported that they were facing some difficulty in accessing food (81%), earning money (90%), and getting routine medical care (73%) during the COVID-19 pandemic.

**Table 1:** Characteristics of the participants (N = 864)

Characteristics	n	%
Age (year)		
60–69	625	72
> = 70	239	28
Sex		
Male	486	56
Female	378	44
Marital status		
Currently Married	683	79
Without partner	181	21
Formal schooling		
No formal schooling	769	89
Having formal schooling	95	11
Household size		
≤ 4	372	43
> 4	492	57
Living arrangement		
Living with family members/others	782	90
Living alone	82	10
Family monthly income (BDT)		
Living on aid	580	67
Have some additional income*	220	33
Current occupation		
Employed	94	11
Unemployed/retired	770	89
Walking distance to the nearest health centre		
< 30 min	592	69
≥ 30 min	272	31
Currently suffering from any non-communicable chronic diseases (NCDs)		
No	431	50
Yes	433	50
Level of physical activity		
Regular at least 2–4 h in a week	295	34
None/Sedentary	569	66
Feeling concerned about COVID-19		
Not concerned	169	20
Somewhat to very concern	695	80
Feeling overwhelmed by COVID-19		
No	190	22
Yes	674	78
Close friend or family member previously diagnosed with COVID-19		
No/Not sure	795	92
Yes	69	8
Frequency of communication during COVID-19		
More than or same as previous	613	71
Less often than previous	251	29
Difficulty of obtaining food during COVID-19		
No difficulty	163	19
Some difficulty	701	81
Difficulty in earning money during COVID-19		
No difficulty	83	9
Some difficulty	781	91
Difficulty of getting routine medical care during COVID-19		
No difficulty	234	27
Some difficulty	630	73

^*^Those who had some additional income other than aid, all of them earned lower than the World Bank defined lower poverty line of USD 3.20 a day

### Prevalence of COVID-19-Related Anxiety and Perceived Stress

Data from the Coronavirus Anxiety Scale (CAS) are presented in Table 2, and the prevalence of perceived stress on Perceived Stress Scale (PSS) is presented in Table 3. The overall prevalence of COVID-19-related anxiety was found to be 68% among the participants. The individual percentage of people who were affirmative to the different items of the CAS was around 50% in each item except that of the last item which was a bit lower (42%). Meanwhile, the overall prevalence of perceived stress was found to be 93% and individual percentage of people who were affirmative to different items of the PSS was more than 70% in each item (Table 3). However, no significant difference was observed in percentage of participants having perceived stress in terms of having COVID-19 anxiety or not (94% versus 92%) (data not shown).

**Table 2:** Prevalence of COVID-19-related anxiety (N = 864)

	n	%
I felt dizzy, lightheaded, or faint, when I read or listened to news about the coronavirus	435	50
I had trouble falling or staying asleep because I was thinking about the coronavirus	477	55
I felt paralyzed or frozen when I thought about or was exposed to information about the coronavirus	438	51
I lost interest in eating when I thought about or was exposed to information about the coronavirus	445	52
I felt nauseous or had stomach problems when I thought about or was exposed to information about the coronavirus	359	42
Overall prevalence of anxiety	591	68

**Table 3:** Prevalence of perceived stress (N = 864)

	n	%
In the last month, how often have you been upset because of something that happed unexpectedly?	622	72
In the last month, how often have you felt that you were unable to control the important events in your life?	635	74
In the last month, how often have you felt nervous and stressed?	629	73
In the last month, how often have you felt confident about your ability to handle personal problems?	659	76
In the last month, how often have you felt that things were going your way?	613	71
In the last month, how often have you found that you could not cope with all the things that you should do?	655	76
In the last month, how often have you been able to control irritations in your life?	657	76
In the last month, how often have you felt that you were on top of things?	646	75
In the last month, how often have you been angered because of things that happen and were uncontrolled?	609	70
In the last month, how often have you felt difficulties were piling up so high that you cannot overcome?	648	75
Overall prevalence of stress	804	93

### Factors Associated with COVID-19-related Anxiety and Perceived Stress

We performed two separate multiple linear regression models to explore the factors associated with COVID-19-related anxiety and perceived stress and results are presented in Table 4 and Table 5. The model multicollinearity diagnosis results for COVID-19-related anxiety and perceived stress are presented separately in the supplementary Tables 1, 2, respectively. VIF values less than 10 for each variable indicate the absence of multicollinearity.

**Table 4:** Factors associated with COVID-19-related anxiety among the participants (N = 864)

Characteristics	β^1^	*P*	95%CI
Sex			
Male	Ref		
Female	0.48	0.139	-0.16, 1.11
Marital status			
Currently married	Ref		
Without partner	0.79	**0.037**	0.05, 1.53
Formal schooling			
No formal schooling	Ref		
Having formal schooling	0.84	0.140	-0.28, 1.95
Household size			
≤ 4	Ref		
> 4	-0.55	0.071	-1.15, 0.05
Current occupation			
Employed	Ref		
Unemployed/retired	-2.36	** < 0.001**	-3.47, -1.24
Level of physical activity			
Regular at least 2–4 h per week	Ref		
None/Sedentary	1.31	** < 0.001**	0.65, 1.97
Feeling concerned about COVID-19			
Hardly	Ref		
Sometimes/often	1.89	** < 0.001**	1.22, 2.57
Close friend or family member previously diagnosed with COVID-19			
No/Not sure	Ref		
Yes	2.44	** < 0.001**	1.17, 3.72
Difficulty in getting food during COVID-19			
No difficulty	Ref		
Some difficulty	1.56	** < 0.001**	0.74, 2.39
Difficulty of getting routine medical care during COVID-19			
No difficulty	Ref		
Some difficulty	2.66	** < 0.001**	1.88, 3.44

^1^We performed a multiple linear regression model to explore the factors associated with COVID-19-related anxiety; adjusted models included all the variables listed in Table 4

R^2^: 0.29

**Table 5:** Factors associated with perceived stress among the participants (N = 864)

Characteristics	β^1^	*P*	95%CI
Marital status			
Currently married	Ref		
Without partner	1.64	**0.005**	0.50, 2.78
Household size			
≤ 4	Ref		
> 4	− 0.72	0.127	− 1.65, 0.21
Current occupation			
Employed	Ref		
Unemployed/retired	− 2.38	**0.001**	− 3,83, -0.92
Feeling overwhelmed by COVID-19			
Hardly	Ref		
Sometimes/often	2.20	** < 0.001**	1.05, 3.36
Frequency of communication during COVID-19			
More than or same as previous	Ref		
Less often than previous	− 2.81	** < 0.001**	− 3.88, − 1.75
Difficulty in getting food during COVID-19			
No difficulty	Ref		
Some difficulty	1.12	0.158	− 0.43, 2.66
Experiencing COVID-19-related anxiety			
No	Ref		
Yes	2.15	**0.001**	0.85, 3.45

^1^We performed a multiple linear regression model to explore the factors associated with perceived stress; adjusted models included all the variables listed in Table 5

R^2^: 0.33

The factors associated with COVID-19-related anxiety reveled in the adjusted regression model are presented in Table 4. The average COVID-19-related anxiety score was expected to be significantly higher (p-value < 0.001) among participants who did not engage in regular physical activity, compared to those who were engaged in at least 2–4 h per week of regular physical activity. Similarly, the average COVID-19-related anxiety score was expected to be significantly higher among the participants who were sometimes or very often concerned about COVID-19 (p-value < 0.001), whose close friends or family members were previously diagnosed with COVID-19 (p-value < 0.001), who had some difficulty in getting food during COVID-19 (p-value < 0.001), and who felt some difficulty of getting routine medical care during COVID-19 pandemic (p-value < 0.001) compared to their counterparts. On the other hand, the average COVID-19-related anxiety score was expected to be significantly lower among those who were without a partner (p-value = 0.037) and who were currently unemployed or retired (p-value < 0.001) compared to their respective counterparts.

The factors associated with perceived stress revealed in the adjusted regression model are presented in Table 5. The average perceived stress score was expected to be significantly higher among participants who were without a partner (p-value = 0.005), who were sometimes to very often feeling overwhelmed by COVID-19 (p-value < 0.001) and who experienced COVID-19-related anxiety (p-value = 0.001) compared to their counterparts. On the other hand, the average perceived stress score was expected to be significantly lower among participants who were currently unemployed or retired (p-value = 0.001), and whose frequency of communication during COVID-19 was less than previous (p-value < 0.001) compared to their respective counterparts.

## Discussion

The current study found that the prevalence of COVID-19-related anxiety and perceived stress was 68% and 93%, respectively. This study also revealed that the average COVID-19-related anxiety score was expected to be significantly higher among those who were physically inactive, was concerned about COVID-19, had close friend or family members diagnosed with COVID-19, and had some difficulty in getting food and routine medical care during the pandemic, compared to their respective counterparts. Meanwhile, the participants without partners, who were feeling overwhelmed by COVID-19, and who experienced COVID-19-related anxiety during the pandemic expected to have significantly higher average perceived stress score.

This study reported a very high prevalence of COVID-19-related anxiety and perceived stress (68% and 93% respectively) among the older adults residing in the Rohingya refugee camp. Poor-socioeconomic conditions, previous traumatic experiences, ongoing risks in their life, uncertainties, and poor access to health care services during the COVID-19 pandemic might have resulted higher level of COVID-19-related anxiety and perceived stress among the participants (Limon et al., 2020; Mistry et al., 2021b, c). We did not find any study conducted in the refugee settings exploring COVID-19 related anxiety and stress using the scales used in the current study to compare with. However, a few studies in other refugee setting used different scales and reported a relatively lower level of COVID-19 related anxiety and stress. For example, a cross-sectional study conducted on migrant returnees (mean age 26 years) in quarantine in Ethiopia found that 48.9% had anxiety symptoms, and more than one-third of the participants (35.6%) had encountered stress (Habtamu et al., 2021). Another study conducted during the COVID-19 pandemic on Bhutanese and Burmese refugees (aged 30–50 years) showed that 68.8% of the participants had stress (Zhang et al., 2022). While these studies were focused on adult population, an Ethiopian study showed a 68.7% prevalence of anxiety among older adults during the COVID-19 pandemic (Jemal et al., 2021). Several factors may explain the differences in anxiety and stress levels among participants in the above studies and those in the current study. They include the usage of different measurement tools, the age range of the study population, pandemic and pre-pandemic condition, socio-cultural variations, refugee settings and other factors affecting anxiety and stress levels.

We found that both COVID-19-related anxiety and perceived stress was higher among the participants who lost their partners. Immediate partner could play a crucial role providing mental support to the fellow partner, particularly during an emergency like COVID-19 pandemic when the support from outside is limited (Jiang et al., 2022; Vowels et al., 2021). In absence of the partner, the participants would have found it difficult to deal with their emotions related to the overwhelming fear associated with COIVD-19 pandemic (Mistry et al., 2021a, b, c, d; Quadros et al., 2021), resulting higher level of COVID-19-related anxiety and perceived stress. We also found that COVID-19-related anxiety and perceived stress was significantly higher among the participants who were feeling concerned/overwhelmed by COVID-19 pandemic. This is also mediated through COVID-19-related fear which resulted a feeling of concern or overwhelm of the pandemic among the participants (Mistry et al., 2021d; Yadav et al., 2021). Previous research conducted among the older adults also documented that worriness related to COVID-19 pandemic resulted in adverse mental health conditions (Khalaf et al., 2022; Webb & Chen, 2022). In line with this, it was revealed that COVID-19-related anxiety was higher among those whose close friends or family members were previously diagnosed with COVID-19. Naturally, a known incident of COVID-19 case within the close connection might have made the participants more fearful of the disease resulting a higher level of anxiety associated with it. Previous research also documented adverse mental health conditions among the people who had their family members diagnosed with COVID-19 (Heesakkers et al., 2022; Tanoue et al., 2020).

Interestingly, we found that COVID-19-related anxiety and perceived stress was significantly lower among the participants who were unemployed or retired. This is probably because the unemployed or retired participants received higher financial assistance from the humanitarian agencies working in the camp during this pandemic (Khan et al., 2020). However, studies conducted among adults in other refugee setting reported that unemployed people faced a higher level of anxiety during this COVID-19 pandemic (Kira et al., 2021; Spiritus-Beerden et al., 2021).

In our study, COVID-19-related anxiety score was higher among the participants who faced difficulties getting food and routine medical care during the pandemic. It is evident that the onset of COVID-19 pandemic resulted in widespread food insecurity and hunger, particularly among the refugee population (Manirambona et al., 2021; UNCHR, 2021) which have caused higher level of anxiety related to the disease. Previous research conducted in the refugee setting also documented that food insecurity has resulted in psychosocial stress (Kaur et al., 2020; Turrini et al., 2017). A study conducted among older Rohingya adults reported that medical services were limited during the COVID-19 pandemic ((Mistry et al., 2021c). Limited health care services, limited access to medicine, difficulties accessing health facilities and fear of getting limited health services from humanitarian organizations might have resulted in higher COVID-19-related anxiety among the participants (Barua & Karia, 2020; Mistry et al., 2021c). This findings are similar to a study conducted among older adults from the Rohingya refugee camps, which showed that difficulties getting food and health care services were associated with older adults’ increased mental health issues during the pandemic (Mistry et al., 2021b).

In our study we found that COVID-19-related anxiety was an independent risk factor of higher perceived stress among the participants during the COVID-19 pandemic. However, in the bi-variate analysis no significant difference was noted in the percentage of participants having perceived stress between the groups having COVID-19-related anxiety or not. This is probably because unlike bi-variate analysis, the regression analysis presents the association after controlling for all the confounding factors. Previous research also highlighted the association between COVID-19-related anxiety and perceived stress (Gallagher et al., 2020; Hu et al., 2021). People often get fearful of COVID-19 pandemic considering its potentials of resulting significant morbidities and mortalities (Chalhoub et al., 2022; Mistry et al., 2021d). Older adults can be particularly fearful thinking that COVID-19 is most lethal among the older adults (Singhal et al., 2021). High level of COVID-19 fear could result in significant anxiety related to COVID-19 which in turn could instigate high level of stress.

Interestingly, findings of the present study also revealed that perceived stress was higher among those who had lesser communication with their family members and friends during the COVID-19 pandemic compared to that of before. This is probably because when people meet their close ones, they tend to discuss more on an important issue like COVID-19 pandemic including its lethality and global reach making them more fearful of it (Mertens et al., 2020) which in turn results higher stress of it. Previous research conducted among the older adults from the Rohingya refugee camp of Bangladesh also revealed that COVID-19 fear was high among those who had lesser communication with their friends and family members during the pandemic (Mistry et al., 2021a).

### Implications for Policy and Practice

Several organizations (International Organization for Migration (IOM), Save the Children International, Action Aid Bangladesh, Caritas Bangladesh, Terre des Hommes) are currently providing mental health and psychosocial support to Rohingya refugees living in Bangladesh (Refugee, 2019). The supports these organizations provided includes community awareness session, need-based individual counseling, podcasting of positive and coping messages in native dialect of Rohingya refugees. However, none of these initiatives particularly addresses the concerns and circumstances of older population and even if available, the acceptability and effectiveness of those initiatives among older adults have not been evaluated. Therefore, our findings suggest the need of evaluation of ongoing interventions and application of those findings in co-design of a people centered interventions that can address the comprehensive psychological determinants of refugee’s health in Bangladesh. Our study’s findings may guide the policymakers, concerned authorities, national and international agencies, and different stakeholders to take appropriate action to address anxiety and stress problems before it poses long term impact on physical and psychological health.

### Strengths and Limitations of the Study

To our knowledge, this is the first study examining COVID-19-related anxiety and perceived stress among older Rohingya refugees during the COVID-19 pandemic. Our study contributed to the limited international literature on anxiety and stress among older Rohingya refugees living in Bangladesh and beyond. Moreover, the study population and study area are unique because the Rohingya camp in Bangladesh is the largest refugee setting in the world. The findings of the study will add important information to the limited existing literature on anxiety and stress among displaced, migrated, and refugee people. Despite these strengths, there are some limitations to mention. First, this study was cross-sectional in design therefore temporal relationship could not be established. Secondly, a limited number of camps and sites were selected for data collection due to restrictions on data collection capacity, which could affect the generalizability of the findings for the entire camp population. Third, this study considered only a few explanatory variables and therefore provides a partial overview of factors that might affect COIVD-19-related anxiety and perceived stress among the participants. Moreover, COVID-19-related anxiety and perceived stress reported in the study was based on self-reported data and not on clinical diagnosis.

## Conclusion

This study found that the prevalence of COVID-19-related anxiety and perceived stress were substantial among the older adults residing in the Rohingya refugee camp in Bangladesh. Various sociodemographic and COVID-19-related factors were associated with this high prevalence of COVID-19-related anxiety and perceived stress that need to be addressed holistically to improve the wellbeing of older adults. Our findings recommend strengthening the existing mental health programs to address growing unmet needs related to anxiety and stress for older adults residing in the Refugee camps. We also highlight the necessity of more comprehensive, integrated, and focused services that would meet the unmet and unattended needs of older adults. Finally, it is critical to provide immediate psychosocial and mental health support for the older Rohingya adults during this COVID-19 pandemic and beyond.

## Supplementary Information

Below is the link to the electronic supplementary material.

Supplementary file1 (DOCX 26 KB)

## Data Availability

All relevant data are within the manuscript and its Supporting Information files.
