# Supplementary material for: Anxiety and Stress Related to COVID-19 Among the Community Dwelling Older Adults Residing in the Largest Refugee Camp of the World
Source: Community Ment Health J. 2023 Mar 6;59(6):1181–92. doi: 10.1007/s10597-023-01101-5 (PMC9988202; doi:10.1007/s10597-023-01101-5)
Supplement: Supplementary file 1 — Supplementary file1 (DOCX 26 KB) [file 10597_2023_1101_MOESM1_ESM.docx]

**Supplementary Table 1. Multicollinearity diagnosis results for anxiety**

| **Variables** | **VIF** | **1/V IF** |
| --- | --- | --- |
| Sex | 1.29 | 0.774299 |
| Marital status | 1.19 | 0.836884 |
| Formal schooling | 1.14 | 0.875996 |
| Household size | 1.05 | 0.953792 |
| Current occupation | 1.17 | 0.856788 |
| Level of physical activity | 1.18 | 0.844787 |
| Feeling concerned about COVID-19 | 1.11 | 0.902682 |
| Close friend or family member diagnosed with COVID-19 | 1.18 | 0.850722 |
| Difficulty in getting food during COVID-19 | 2.30 | 0.435700 |
| Difficulty of getting routine medical care during COVID-19 | 2.21 | 0.452130 |
| **Mean VIF** | **1.38** |  |

**Supplementary Table 2. Multicollinearity diagnosis results for stress**

| **Variables** | **VIF** | **1/V IF** |
| --- | --- | --- |
| Marital status | 1.02 | 0.977013 |
| Current occupation | 1.03 | 0.973968 |
| Feeling overwhelmed by COVID-19 | 1.05 | 0.953128 |
| Frequency of communication during COVID-19 | 1.10 | 0.907821 |
| Difficulty in getting food during COVID-19 | 1.06 | 0.942929 |
| **Mean VIF** | **1.05** |  |
